# A New Furofuran Lignan Diglycoside and Other Secondary Metabolites from the Antidepressant Extract of *Castilleja tenuiflora* Benth

**DOI:** 10.3390/molecules200713127

**Published:** 2015-07-21

**Authors:** Maribel Herrera-Ruiz, Ricardo López-Rodríguez, Gabriela Trejo-Tapia, Blanca Eda Domínguez-Mendoza, Manases González-Cortazar, Jaime Tortoriello, Alejandro Zamilpa

**Affiliations:** 1 Centro de Investigación Biomédica del Sur, Instituto Mexicano del Seguro Social, Argentina No. 1, Col. Centro, Xochitepec, Morelos 62790, Mexico; E-Mails: cibis_herj@yahoo.com.mx (M.H.-R.); richard_lorr@hotmail.com (R.L.-R.); gmanases@hotmail.com (M.G.-C.); jtortora2@yahoo.es (J.T.); 2 Departamento de Biotecnología, Centro de Desarrollo de Productos Bióticos, Instituto Politécnico Nacional, P.O. Box 24, Yautepec, Morelos 62730, Mexico; E-Mail: gttapia@ipn.mx; 3 Laboratorio de RMN, Centro de Investigaciones Químicas, Universidad Autónoma del Estado de Morelos, Av. Universidad 1001, Colonia Chamilpa C.P. Cuernavaca, Morelos 62210, Mexico; E-Mail: bed@uaem.mx

**Keywords:** *Castilleja tenuiflora*, furofuran lignans, iridoids, verbascoside, flavonoids

## Abstract

*Castilleja tenuiflora* has been used for the treatment of several Central Nervous System (CNS) diseases. Herein we report the antidepressant activity of the methanol extract from the leaves of this medicinal plant. The oral administration of MeOH extract (500 mg/kg) induced a significant (*p* < 0.05) decrement of the immobility parameter on Forced Swimming Test (FST) and an increment in the latency and duration of the hypnosis, induced by administration of sodium pentobarbital (Pbi, 40 mg/kg, *i.p.*). Chemical analysis of this antidepressant extract allowed the isolation of (+)-piperitol-4-*O*-xylopyranosyl-(1→6)-*O*-glucopyranoside. This new furofuran lignan diglycoside was named tenuifloroside (**1**) and its complete chemical structure elucidation on the basis of 1D and 2D NMR spectra analysis of the natural compound **1** and its peracetylated derivative **1a** is described. This compound was found together with two flavones—apigenin and luteolin 5-methyl ether—a phenylethanoid—verbascoside—and three iridoids—geniposide, caryoptoside and aucubin. All these compounds were purified by successive normal and reverse phase column chromatography. Tenuifloroside, caryoptoside and luteolin 5-methyl ether were isolated from *Castilleja* genus for the first time. These findings demonstrate that *C. tenuiflora* methanol extract has beneficial effect on depressive behaviors, and the knowledge of its chemical constitution allows us to propose a new standardized treatment for future investigations of this species in depressive illness.

## 1. Introduction

*Castilleja tenuiflora* Benth is a small perennial shrub belonging to the Orobanchaceae family and is distributed in mountainous areas the Southern United States and Mexico [1]. This plant is one of the 220 species comprised in the *Castilleja* genus and is commonly known as “garañona”, “cola de borrego” or “hierba del cancer” and used in Mexican Traditional Medicine for the treatment of conditions associated with cancer symptomatology as well as for the treatment of coughs, dysentery, nausea, vomiting, hepatic, gastrointestinal and nervous disorders [2,3,4,5]. The anti-inflammatory, antioxidant, cytotoxic and anti-ulcerogenic activities of different extracts from *C. tenuiflora* have been reported [6,7,8]. Previous chemical studies of this species have shown the presence of flavonoids (apigenin, quercetin glycosides), glycosylated iridoids (aucubin, bartsioside, mussaenosidic acid, geniposidic acid, methyl 8-*epi*-loganin, geniposide, shanzhiside) and phenylethanoid derivatives (verbascoside and isoverbascoside) [8,9,10,11]. Considering that *C. tenuiflora* has been used for the treatment of nervous disorders, this work was focused in demonstrating the antidepressant and sedative effects of *C. tenuiflora*. Chemical analysis of this plant allowed the isolation of a new furofuran lignan diglycoside whose structural elucidation is described. Other secondary metabolites isolated from this antidepressant extract were two flavones, a phenylethanoid and three iridoids.

## 2. Results and Discussion

**Figure 1:**
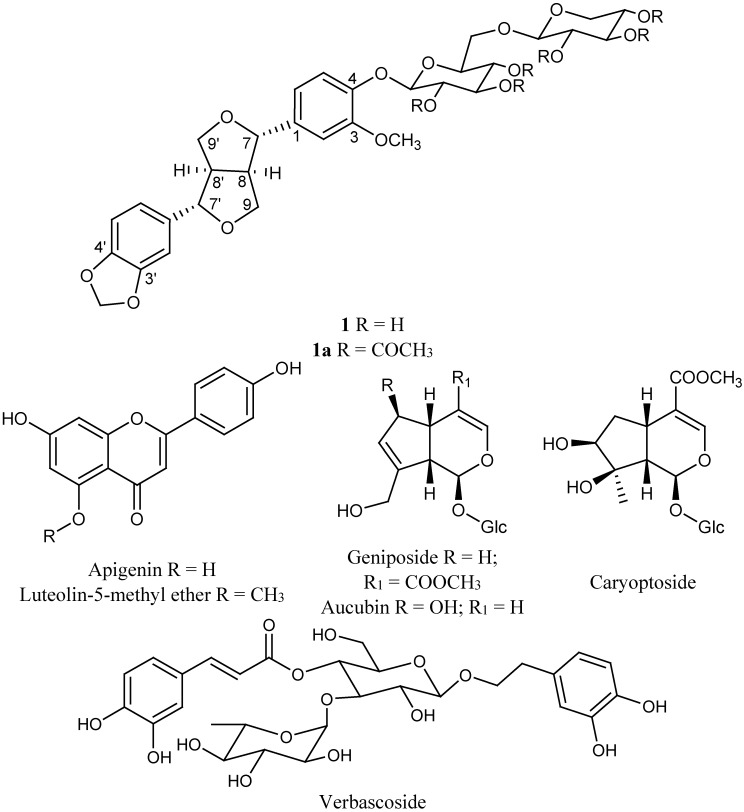
Chemical constituents from methanol extract of *C. tenuiflora* leaves.

**Figure 2:**
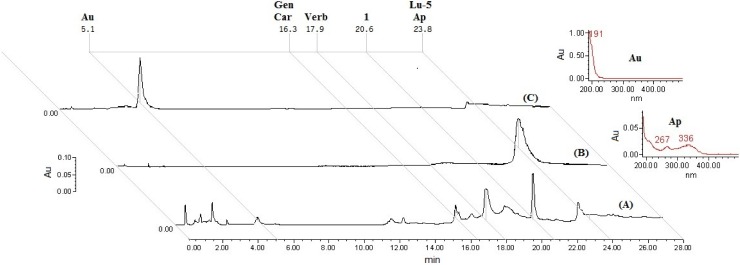
Chromatographic profiles of the methanol extract (**A**), apigenin reference (**B**), aucubin reference (**C**). Chromatograms were developed at the UV wavelength of 205 (**A**,**C**) and 340 (**B**) nm. The marked peaks (Rt = 5.1, 16.3, 17.9, 20.6 and 23.8 min) are assigned to aucubin (**Au**), geniposide (**Gen**), caryoptoside (**Car**), tenuifloroside (**1**), apigenin (**Ap**) and Luteolin 5-methyl ether (**Lu-5**) respectively.

**Figure 3:**
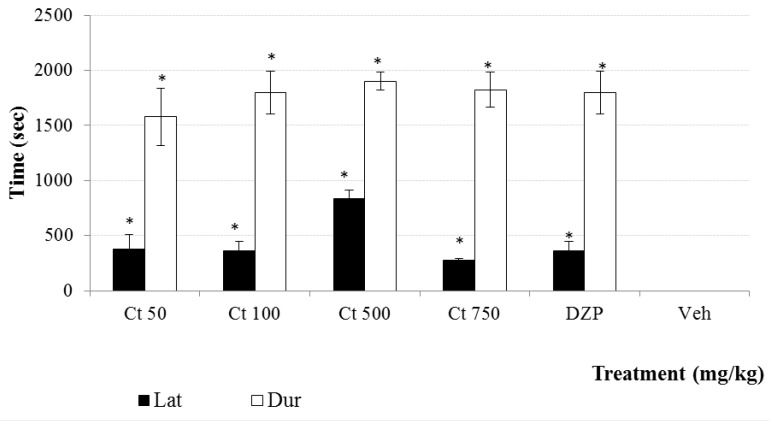
Effect produced by different doses (50, 100, 500 and 750 mg/kg, *p.o.*) of Ct extract from *C. tenuiflora* on the latency and duration of hypnosis induced by sodium pentobarbital (40 mg/kg, sub hypnotic dose). Results are presented as means ± SD. * *p* < 0.05, as compared with the control group. (ANOVA one way following by Dunnet test); *n* = 6 mice per group. Lat = latency, Dur = duration, DZP = Diazepam, Veh = Tween 20, 1.0%.

The methanol extraction of 1.2 kg of dry plant material produced 187.2 g of extract (15.6%). This whole extract was used for biological activity and chemical analysis and its study allowed the identification of a new furofuran lignan **1**. The known compounds apigenin, luteolin 5-methyl ether, verbascoside, geniposide, caryoptoside and aucubin were also identified in this extract. Compound **1** and verbascoside were the major constituents, while apigenin and luteolin 5-methyl ether were the minor compounds (Figure 1 and Figure 2). The methanol extract (Ct 50, 100, 500 and 750 mg/kg, *p.o.*) produced a significant (*p* < 0.05) increment in the latency and duration of the hypnosis induced by administration of sodium pentobarbital (Pbi, 40 g/kg, *i.p.*) in respect to the control group (vehicle, Tween 20 1% solution) (Figure 3). A similar effect was observed with the sedative reference diazepam (DZP, 1.0 mg/kg, *i.p.*). Sedation is a depression state of the CNS produced by the administration of sedative drugs in which alertness and motor activity are reduced. In animal experiments the term “hypnotic” has been related with a deep state of central depression after drugs administration with association of loss of muscle tone and righting reflex. This parameter is regularly evaluated for pharmacological tests enhancement based on barbiturate-induced hypnotic state or other sedative agents [12,13,14].

Previous reports have described that apigenin, verbascoside, geniposide and aucubin isolated from several medicinal plants show different neuroprotective effects. For instance, verbascoside is capable of inhibiting the glutamate-induced intracellular Ca^2+^ influx with overproduction of nitric oxide and reduction of reactive oxygen species. Geniposide had a multifaceted neuroprotective effect in an Alzheimer mouse model. Furthermore apigenin modulates GABAergic and glutamatergic transmission in culture cortical neurons and was able to increase the sleep time induced by pentobarbital [15,16,17,18].

**Figure 4:**
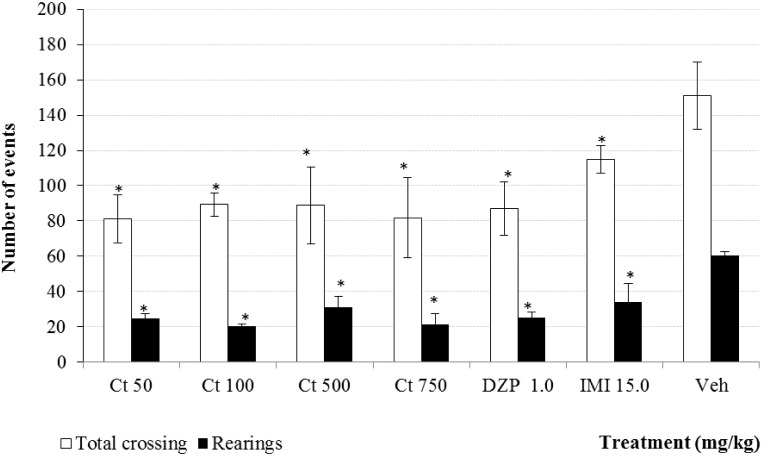
Effect produced by different doses (50, 100, 500 and 750 mg/kg, *p.o.*) of Ct extract from *C. tenuiflora* (Ct) on spontaneous motor parameters: total crossing and rearings in the OFT. The results are presented as means ± SD. * *p* < 0.05, as compared with the control group. (ANOVA one way following by Dunnet test); *n* = 6 mice per group. DZP = Diazepam, IMI = Imipramine, Veh = Tween 20, 1.0%.

The Open Field Test model (OFT) is a paradigm used to evaluate the sedative effect of drugs on general behavior. This test is used for measuring the level of nervous excitability, thus substances that diminish this motor behavior correlate with a central inhibition [19,20]. In this work the administration of different doses of Ct induced a significant decrement in the parameters of spontaneous motor activity like rearing and total crossing. This observation was similar with the sedative drug diazepam (DZP, 1.0 mg/kg, Figure 4). Imipramine (IMI) induced a decrease in the parameters of spontaneous motor activity. These results indicate that Ct has a depressant effect on the central nervous system which is in accord with the enhancing activity of the hypnotic effect of pentobarbital. This medicinal plant is traditionally used for calming nervous conditions and for the treatment of anger control. Here both sedative and hypnotic effects were demonstrated. Previous reports showed that verbascoside (3 mg/kg, *p.o.*) produces sedative effect induced in the OFT [21].

**Figure 5:**
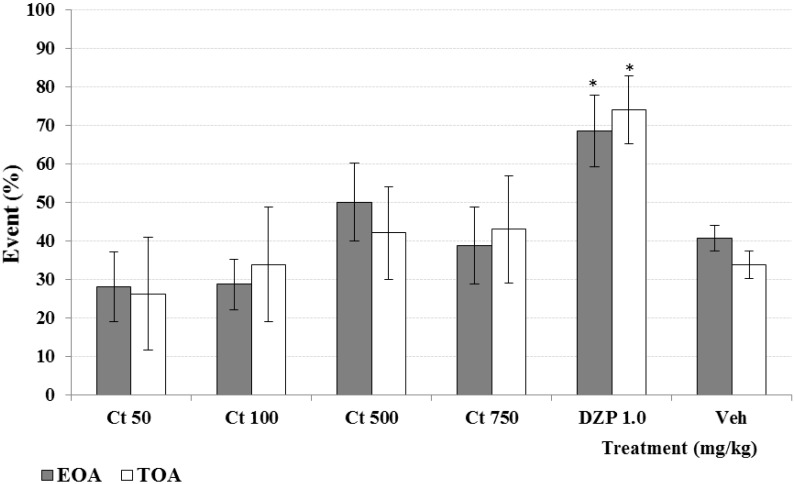
Effect produced by different doses (50, 100, 500 and 750 mg/kg, *p.o.*) of Ct extract from *C. tenuiflora* (Ct) on percentage of entries (EOA) and time (TOA) spent by mice on open arms in the EPM. The results are presented as means ± SD. * *p* < 0.05, as compared with the control group. (ANOVA one way following by Dunnet test); *n* = 6 mice per group. DZP = Diazepam, Veh = Tween 20, 1.0%.

In the plus maze test, no dose of *C. tenuiflora* extract showed anxiolytic activity (Figure 5). Validation of this model is based on the use of drugs as benzodiazepines which increase the time and number of entries in the open arms of the maze as signal of diminution of fear (anxiety).

**Figure 6:**
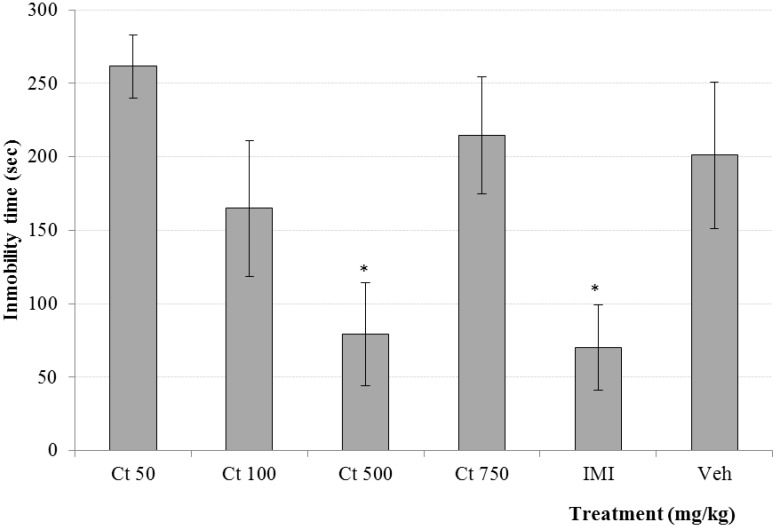
Effect produced by different doses (50, 100, 500 and 750 mg/kg, *p.o.*) of Ct extract from *C. tenuiflora* (Ct) on immobility time in the FST. The results are presented as means ± SD. * *p* < 0.05, as compared with the control group. (ANOVA one way following by Dunnet test); *n* = 6 mice per group. IMI = Imipramine, Veh = Tween 20, 1.0%.

Antidepressant clinical drugs such as IMI or fluoxetine among other drugs have been tested in Forced Swimming Test model on mice (FST) inducing an increment in the immobility parameter when mice are forced to swim in a water cylinder [22]. Under this condition, the oral administration of Ct (500 mg/kg, Figure 6) induced a significant (*p* < 0.05) decrement of the immobility parameter while minor doses (50 and 100 mg/kg) did not produce any change in this parameter in comparison with the vehicle group. When the treatment was evaluated at 750 mg/kg the effect disappears. The result of this effect is in U-Shape, which is not surprising because there are other studies that show that medicinal species provoke different activities in this form, such as *Hypericum perforatum* (30–250 mg/kg) or the Asian herbal remedy *Nidrakar baty* (100–400 mg/kg). Both medicinal plants induce antidepressant effect in this way in FST [23,24]. On the other hand, *Carica papaya* showed anxiolytic effect when it was evaluated at 100 mg/Kg but it was inactive at doses of 50 and 400 mg/kg [25]. An inverted U-shape activity was observed with the ethanol extract from *Glycyrrhiza glabra* when it was evaluatated (10, 20 and 30 mg/kg) in the elevated plus maze test [26]. In relation with anticonvulsant activity of alcoholic extracts (100–1000 mg/kg) from *Saussurea lapa*, positive effects were observed at 100 and 1000 mg/kg whereas the dose of 300 mg/kg was inactive [27]. All these studies concluded that chemical complexity of the integrate extracts may result in this kind of biphasic responses. In the pharmacological literature, these facts are explained by the presence of different receptors which mediate opposite effects with high and low agonist affinity in biological, toxicological and medicinal analysis [28]. The chemical complexity of the Ct extract may result in this kind of pharmacological response. The antidepressant activity of many substances, on the FST could be the result of the false-positive, due to an increment on motor behavior of mice. In this case, it is necessary to carry out other behavior test, such as OFT. Above was shown the effect of Ct on that pharmacological test, where it was observed that this extract induced a decrement of the motor parameters similar to DZP and IMI. Such results are indicative that the antidepressant effect of 500 mg/kg of Ct, has a similar behavior of IMI which decreases the total crossing and rearings on the OFT [29]. In this case, all doses of CtME induced a similar depressive effect on CNS which can be attributed to the chemical complexity of the crude extract. In this way, the antidepressant activity of Ct extract does not depend on the stimulation of the CNS.

**Figure 7:**
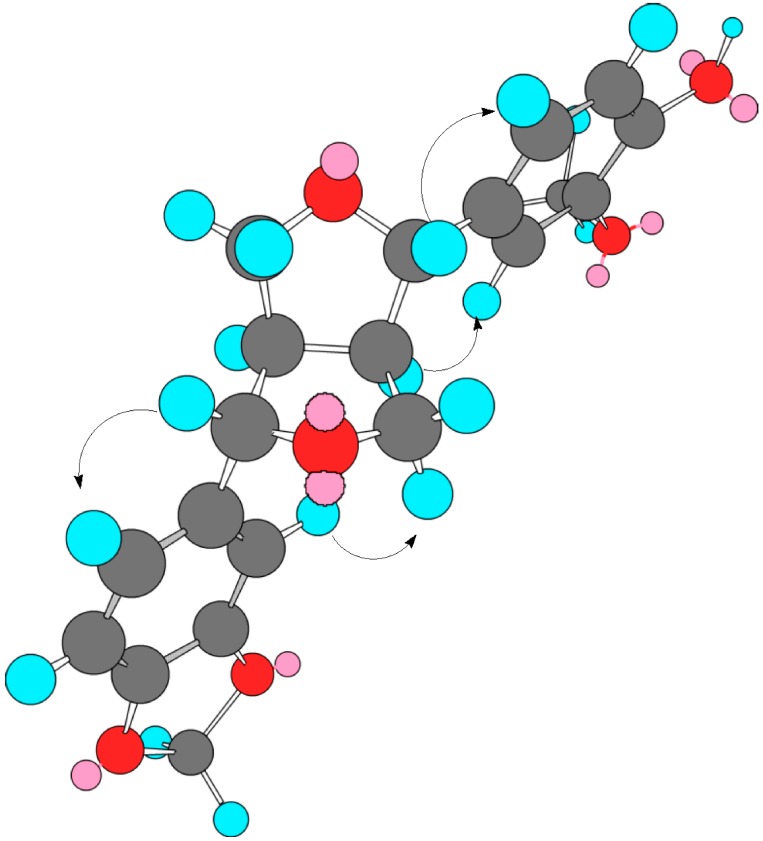
A 3D structure of the furofuran lignan aglycone obtained by computer imaging (Chem Draw 3D) showing the major interactions observed in the NOESY experiment.

**Figure 8:**
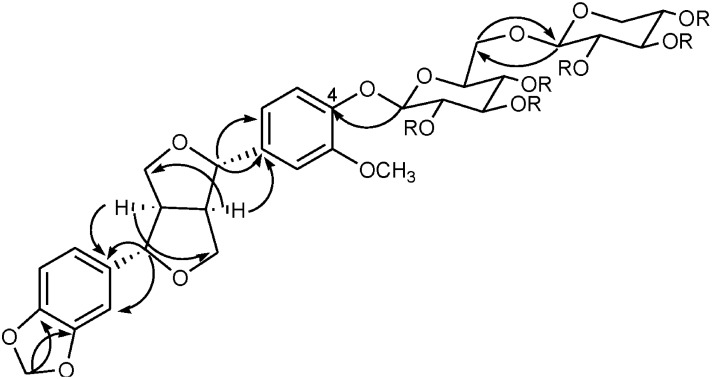
Key HMBC correlations of compounds **1** and **1a** (Recorded on Varian INOVA-400).

Successive normal and reverse phase chromatography of the Ct extract allowed the isolation and purification of a new furofuran lignan diglycoside which was denominated tenuifloroside (**1**, 125 mg). Other six known compounds, namely apigenin (6 mg), luteolin 5-methyl ether (5 mg), verbascoside (72 mg), geniposide (18 mg), caryoptoside (14 mg) and aucubin (11 mg) were also identified (Figure 1). The structure of the new compound was established using spectrometric (FABMS) analysis and spectroscopic (^1^H- and ^13^C-NMR, COSY, TOCSY, NOESY, HSQC, HMBC) experiments of the natural occurring compound **1** and its hexaacetate derivative **1a**. The chemical structure of the known compounds was established by comparison of their spectroscopic data (UV and NMR) with published reports and by direct HPLC comparison with commercial references. Caryoptoside has not been previously described for this species. Compound **1** was isolated as white amorphous powder and gave an ion peak at *m*/*z* 673 [M + Na]^+^ in the positive FABMS, corresponding to a molecular formula C_31_H_38_O_15_ and this was supported by HR-ESI-MS [*m*/*z* 673.2139 [M + Na]^+^]. The ^1^H and ^13^C spectra of compound **1** suggested certain common structural features attributable to a sugar moiety, two 1,3,4-trisubstituted phenyls and a furofuran skeleton. Tenuifloroside (**1**, 117 mg) revealed strong UV and IR typical absorption bands typical of furofuran lignans (λ_max_ = 201, 228, 282 *n*m and 3392, 2910, 1630, 1514 cm^−1^ respectively) [30]. The hexaacetate derivative **1a** gave an ion peak at *m*/*z* 903.3210 [M + H]^+^ which corresponds to the formula C_43_H_51_O_21_. Assignment of all the ^13^C- and ^1^H-NMR signals for each spin systems is as shown in Table 1. Six aromatic protons distributed in two ABX spin systems: δ 7.07 (1H, d, *J* = 8.4 Hz, H-5), 6.93 (1H, d, *J* = 2.0 Hz, H-2), 6.90 (1H, dd, *J* = 8.4, 2.0 Hz, H-6) and 6.84 (1H, d, *J* = 1.56 Hz, H-2′), 6.80 (1H, dd, *J* = 8.0, 1.56, Hz, H-6′) and 6.76 (1H, d, *J* = 8.0 Hz, H-5′). Both methylenedioxy and methoxy groups were placed at δ 5.93 (2H, s) and 3.83 (3H, s) respectively. The eight furofuran skeleton signals displayed chemical shifts at δ 4.77 (1H, d, *J* = 4.8 Hz, H-7′), 4.69 (1H, d, *J* = 5.2 Hz, H-7), 4.26 (1H, dd, *J* = 9.2, 6.8 Hz, H-9a), 4.21 (1H, dd, *J* = 9.2, 6.8 Hz, H-9a′), 3.88 (1H, dd, *J* = 4.0, 4.0 Hz, H-9b), 3.86 (1H, dd, *J* = 4.0, 4.0 Hz, H-9b′′), 3.07–3.12 (1H, m, H-8′), 3.02–3.06 (1H, m, H-8) which corresponds to a di-equatorial furofuran series [31]. Diglycoside moiety is constituted by xylopyranose and glucopyranose monosaccharides. COSY, TOCSY and HSQC-TOCSY experiments were used to identify all vicinal spin system of each monosaccharide as follows: 5.25 (1H, dd, *J* = 8.0,9.6 Hz, Glc-2), 5.22 (1H, dd, *J* = 9.6, 9.6 Hz, Glc-3), 5.09 (1H, dd, *J* = 8.4, 8.4 Hz, Xyl-3), 4.99 (1H, dd, *J* = 9.6, 9.6 Hz, Glc-4), 4.92 (1H, d, *J* = 8.0 Hz, Glc-1), 4.91 (1H, ddd, *J* = 8.4, 8.4, 4.8 Hz, Xyl-4), 4.87 (1H, dd, *J* = 8.4, 6.8 Hz, Xyl-2), 4.53 (1H, d, *J* = 6.8 Hz, Xyl-1), 4.09 (1H, dd, *J* = 11.6, 8.4 Hz, Xyl-5α), 3.80–3.85 (1H, m, Glc-6α), 3.74(1H, ddd, *J* = 9.6, 6.6, 2.0 Hz, Glc-5), 3.66 (1H, dd, *J* = 11.2, 6.6 Hz, Glc-6β), 3.26 (1H, dd, *J* = 11.6, 4.8 Hz, Xyl-5β). The β-glucopyranose and β-xylopyranose nature was determined by the all *trans* diaxial coupling constants (Table 1). ^13^C-NMR and HMQC-TOCSY, HMBC experiments allowed the assignments of each carbon signals: Twelve aromatic carbons were placed at: δ 150.65(C-3), 148.13 (C-4′), 147.28 (C-3′), 145.99 (C-4), 137 (C-1′), 135.15 (C-1), 119.60 (C-6′), 119.22 (C-5), 118.73 (C-6), 110.58 (C-2), 108.35 (C-5′), 106.68 (C-2′) which corresponds with two phenyl spin systems. One dioxymethylene carbon was placed at δ 101.26, four oxygenated methylene carbons were placed at δ 86.01(C-7′), 85.65(C-7), 72.08 (C-9), 71.82 (C-9′) and one methoxy group at δ 56.28. The signals corresponding to the furofuran, were very similar to those of the equatorial-equatorial furofuran in (+)-piperitol-4-*O*-β-d-glucopyranoside [32]. On the other hand, chemical shift differences for the benzylic protons at δ 4.77 (1H, d, *J* = 4.8 Hz, H-7′), 4.69 (1H, d, *J* = 5.2 Hz, H-7), and carbons placed at δ 86.01 (C-7′), 85.65(C-7) were observed when compound **1a** was compared with the *epi*-series furofuran glucosylated lignan: styraxjaponoside C and with diaxial series (–)-pinoresinol [33,34]. This *eq*-*eq* orientation of the phenyl groups was corroborated by the NOE interaction between H-6 placed at δ 6.90 (dd, *J* = 8.0, 2.0 Hz) with H-7 placed at δ 4.77 (d, *J* = 4.8 Hz) and H-6′ placed at δ 6.76 (dd, *J* = 8.0, 1.56 Hz) with H-7′ placed at δ 4.69 (d, *J* = 5.2 Hz) as well as the interaction between H-8′ placed at δ 3.07–3.12 (m) with H-2′ placed at δ 6.84 (d, *J* = 1.56 Hz) observed in a NOESY experiment (Figure 7). All of this indicated that the aglycone of this furofuran lignan corresponds to the (+)-piperitol. In the HMBC experiment the carbon signals placed at δ 148.13 (C-4′) and 147.28 (C-3′) showed a correlation to the proton signal at δ 5.93 (OCH_2_O), carbons assigned to C-1′(δ 137.73), C-2′(δ 106.68) and C-6′(δ 119.60) correlated to the proton signal at δ 4.77 (H-7′), and the carbons signals at δ 135.15 (C-1) and 119.92 (C-6) correlated to the proton signal at δ 4.69 (H-7) (Figure 8). HSQC experiment allowed to identify all carbon signals of the per-acetylated glucopyranosyl and xylopyranosyl systems at δ 101.26 (OCH_2_O ), 100.88 (Glc-1′′), 100.53 (Xyl-1′′′), 86.01, 85.65), 73.97 (Glc-5′′), 72.67 (Glc-′′), 72.08 (C-9), 71.82 (C-9′), 71.37 (Glc-3′′), 71.29 (Xyl-3′′′), 70.66 (Xyl-2′′′), 68.96 (Xyl-4′′′), 68.89 (Glc-4′′), 67.60 (Glc-6′′), 62.11 (Xyl-5′′′). The interglycosidic linkage of these sugar units was derived from HMBC correlations. A cross-peak due to long range correlations between the anomeric proton of xylose at 4.53 (1H, d, *J* = 6.8 Hz) and C-6 of the glucopyranosyl unit at 67.60 as well as the cross-peak due to long range correlations between the anomeric carbon of xylose at δ 100.58 whit H-6α at δ 3.80–3.85 (1H, m), and H-6 β at 3.66 (1H, dd, *J* = 11.2, 6.8 Hz) indicated that xylopyranose was linked in C-6 of the glucopyranose. The position of the sugar residue in **1a**, was defined to be at C-4 due to long range correlation observed between the oxygenated aromatic carbon C-4 at δ 145.99 with the anomeric proton at δ 4.92 (1H, d, *J* = 8.0 Hz) of the glucopyranose sugar unit. Based on all these evidences the natural product was identified as (+)-piperitol-4-*O*-β-xylopyranosyl-(1-6)-β-glucopyranoside which was denominated tenuifloroside (**1**). The other known compounds were identified by comparison of their spectroscopic data with those previously reported: luteolin 5-methyl ether, verbascoside, geniposide and caryoptoside as well as the chromatographic profile compared to the commercial references: apigenin, and aucubin [9,10,35,36]. Caryoptoside has not been previously isolated from *Castilleja* genus. The major compound in the methanolic extract was verbascoside while the minor compound corresponds to apigenin (Figure 2).

**Table 1:** ^1^H (400 MHz) and ^13^C (100 MHz) NMR data for compounds **1** (DMSO-*d*_6_) and **1a** (CDCl_3_).

		(1a)		Tenuifloroside (1)	
Position		δ_C_, Type	δ_H_ (*J* in Hz)	δ_C_	δ_H_ (*J* in Hz)
	1	135.1, C	-	135.59	-
	2	110.5, CH	6.93 (1H, d, 2.0)	110.87	6.91 (1H, d, 1.6)
	3	150.6, C	-	149.24	-
	4	145.9, C	-	146.20	-
	5	119.2, CH	7.07 (1H, d, 8.4)	115.88	7.07 (1H, d, 8.4)
	6	118.7, CH	6.90 (1H, dd, 8.4, 2.0)	118.82	6.82 (1H, dd, 8.4, 1.6)
	7	85.6, CH	4.77 (1H, d, 4.8)	85.40	4.6 (1H, d, 4.8)
	8	54.2, CH	3.02–3.06 (1H, m)	54.13	2.97 (1H, m)
	9	72.08, CH_2_	4.26 (1H, dd, 9.2, 6.8)	71.45	4.13 (1H, m)
			3.88 (1H, dd, 4.0, 4.0)		3.74 (1H, dd, 4.0, 4.0)
	1′	137.7, C		135.91	-
	2′	106.6, CH	6.84 (1H, d, 1.56)	107.00	6.89 (1H, d, 1.2)
	3′	147.2, C	-	146.91	-
	4′	148.1, C	-	147.84	-
	5′	108.3, CH	6.76 (1H, d, 8.0)	108.47	6.83 (1H, d, 8.4)
	6′	119.6, CH	6.80 (1H, dd, 8.0, 1.56,)	118.85	6.83 (1H, 8.4, 1.2)
	7′	86.01, CH	4.69 (1H, d, 5.2)	85.30	4.6 (1H, d, 4.8)
	8′	54.46, CH	3.07–3.12 (1H, m)	54.17	2.97 (1H, m)
	9′	71.8, CH_2_	4.21 (1H, dd, 9.2, 6.8)	71.45	4.13 (1H, m)
			3.86 (1H, dd, 4.0, 4.0)		3.74 (1H, dd, 4.0, 4.0)
OCH_3_		56.28	3.83 (3H, s)	56.14	3.74 (3H, s)
OCH_2_O		101.26	5.93 (2H, s)	101.32	5.95 (2H, s)
Glc	1	100.8 CH	4.92 (1H, d, 8.0)	100.62	4.82 (1H, d, 7.6)
	2	72.6, CH	5.25 (1H, dd, 8.0,9.6)	73.59	3.24 (1H, m)
	3	71.3, CH	5.22 (1H, dd, 9.6,9.6)	77.06	3.24 (1H, m)
	4	68.8, CH	4.99 (1H, dd, 9.6, 9.6)	69.98	3.14 (1H, m)
	5	73.97, CH	3.74 (1H, ddd, 9.6, 6.6, 2.0)	76.35	3.49 (1H, m)
	6α	67.60, CH_2_	3.80–3.85 (1H, m)	68.47	3.90 (1H, d br, 10.4)
	6β		3.66 (1H, dd, 11.2, 6.6)		3.53 (1H, dd, 11.6, 6.4)
Xyl	1	100.53, CH	4.53 (1H, d, 6.8)	104.12	4.13(1H, m)
	2	70.66, CH	4.87 (1H, dd, 8.4, 6.8)	73.81	2.93 (1H, m)
	3	71.29, CH	5.09 (1H, dd, 8.4, 8.4)	76.89	3.04 (1H, m)
	4	68.96, CH	4.91 (1H, ddd,8.4,8.4, 4.8)	69.98	3.24 (1H, m)
	5α	62.11, CH_2_	4.09 (1H, dd, 11.6, 8.8)	65.98	2.90 (1H, m)
	5β		3.26 (1H, dd, 11.6, 4.8)		3.64 (1H, dd, 5.6, 11.2)

## 3. Experimental Section

### 3.1. General Information

Optical rotations were measured on a 241 digital polarimeter (PerkinElmer, Waltham, MA, USA) equipped with a sodium lamp (589 nm) and a microcell. UV spectra were obtained on a 2996 photodiode array detector (Waters, Milford, MA, USA). The IR spectrum was recorded on a Tensor 27 FTIR (Bruker, Fremont, CA, USA). All NMR spectra and two-dimensional spectroscopy experiments COSY, TOCSY, HSQC, HMBC were recorded on an INOVA-400 instrument (Varian, Palo Alto, CA, USA) at 400 MHz for ^1^H-NMR spectra in CDCl_3_, CD_3_OD or DMSO-*d*_6_ with tetramethylsilane (TMS) as internal standard. Chemical shifts are reported in δ values. High resolution-electrospray ionization mass spectrometry (HR-ESI-MS) in the positive and negative ion mode was performed using an AX 505 HA (JEOL, Tokio, Japan) mass spectrometer. Analytical TLC was carried out on precoated Merck silica gel 60F_254_ or RP-18F_254_ plates. HPLC separations were performed on a Waters 2695 separations module equipped with a Waters 2996 photodiode array detector and a Supersphere RP-18 (125 × 4 mm) column.

### 3.2. Plant Materials

*Castilleja tenuiflora* was collected at Juchitepec, State of Mexico, Mexico in November 2012. This place is situated at 2800 m.a.s.l. (latitude 19°10 N, longitude 98°92 W). Plants were positively identified as *C.* *tenuiflora* by Rolando Ramírez, M. Sc., Head Curator of the HUMO Herbarium of the Morelos State University (UAEM, Morelos, Mexico). A voucher specimen (HUMO25205) was deposited in the herbarium.

### 3.3. Extraction and Isolation of Chemicals Compounds from MeOH Extract

The whole plant material was air-dried and pulverized with a grinder and extracted with methanol (1.8 kg in 9 L) by maceration at room temperature for 24 h. The liquid extract was filtered using No. 1 Whatman filter paper and concentrated to dryness in a Büchi-490 rotary evaporator (Büchi, Flawil, Switzerland) at 40 °C under low pressure. Final extract (201.6 g) was stored at 4 °C for later chromatographic and pharmacologic analysis. The MeOH extract (Ct, 64 g) was subjected to silica gel column (302 g, 20 × 60 cm) using CH_2_Cl_2_–MeOH gradient system (100:0, 95:5, 90:10, 80:20, 70:30, 60:40, 50:50 and 0:100; 100 mL) to give 67 samples grouped in eight fractions (Fr1-Fr8).

Fraction 2 (0.7 g) was rechromatographed on a silica gel column (30 g, 2 × 30 cm) with CH_2_Cl_2_–CH_3_COCH_3_ gradient system (100:0, 90:10, 80:20, 70:30 and 0:100; 20 mL) to give 15 samples grouped in five fractions (Fr.2-1 to Fr.2-5). Fractions 2-3 (12 mg) displayed the same retention time (23.8 min) and UV spectra (λ_max_ = 213, 267, 335 nm) as a commercial standard of apigenin (**2**, Figure 7). Fraction 4 (3.5 g) was rechromatographed on a silica gel column (80 g, 2 × 30 cm) with a CH_2_Cl_2_–MeOH gradient system (100:0, 95:5, 90:10, 80:20, 70:30, 60:40, 50:50 and 0:100; 30 mL) to afford 45 samples finally grouped in eight fractions (Fr.4-1 to Fr.4-8). Fraction 4-3 contained a white precipitate which was filtered and washed (5 mL of CH_2_Cl_2_) to give a mixture of geniposide and caryoptoside. Fraction 6 (3.5 g) was rechromatographed on a RP-18 silica gel column (35 g, 2 × 30 cm) with a H_2_O–acetonitrile gradient system (100:0, 90:10, 80:20, 70:30, 60:40, and 0:100; 30 mL) giving 28 samples grouped in seven fractions (Fr.6-1 to Fr.6-7). Fraction F6-2 displayed the same retention time (5.1 min) and UV spectra (192 nm) as commercial standard of aucubin (Figure 2). Fraction Fr.6-4 (0.9 g) was purified on a RP-18 silica gel column (10 g, 1.2 × 30 cm) using a H_2_O–acetonitrile gradient system (100:0, 90:10, 80:20, 70:30, 60:40, and 0:100; 10 mL) giving 16 samples grouped in five fractions (Fr.7-1 to Fr.7-5). Fraction Fr.7-4 contained a white solid with identical ^1^H- and ^13^C-NMR data as previously described for verbascoside [10]. Subfraction 6-6 produced a white precipitate which was washed (CH_3_COCH_3_, 10 mL) to afford tenuifloroside (**1**, 125 mg) as an amorphous white residue (MeOH); ${\left[\text{a}\right]}_{\text{D}}^{28}$ + 10.0 (*c* 1.0 H_2_O); UV (λ_max_ from HPLC 201, 228, 282 nm; IR (KBr) ν_max_3392, 2910, 1630, 1514 cm^−1^; ^1^H (DMSO-*d*_6_, 400 MHz) and ^13^C DMSO-*d*_6_, 100 MHz NMR data , see Table 1; ESIMS *m*/*z* 673 [M + Na]^+^; HRESIMS (positive) *m*/*z* [673.2139 [M + Na]+] (calcd for C_31_H_38_O_15_Na).

### 3.4. Acetylation of Compound **1**

Tenuifloroside (**1**, 55 mg) was treated with Ac_2_O (1 mL) and pyridine (0.5 mL) for 2 h under stirring. Extraction with ethylacetate (6.5 mL, three times) after the addition of water (5 mL) into the reaction mixture afforded the peracetate derivative **1a** (42 mg) as an amorphous white residue (MeOH); ${\left[\text{a}\right]}_{\text{D}}^{28}$ + 10.0 (*c* 1.0 H_2_O); IR (KBr) ν_max_ 3392, 2910, 1630, 1514 cm^−1^; CD; ^1^H (DMSO-*d*_6_, 400 MHz) and ^13^C DMSO-*d*_6_, 100 MHz NMR data, see Table 1; ESIMS *m*/*z* 673 [M + Na]^+^; HRESIMS (positive) *m*/*z* [673.2139 [M + Na]+] (calcd for C_31_H_38_O_15_).

### 3.5. HPLC Analysis

Commercial standards of aucubin (>99% HPLC, Fluka, St. Louis, MO, USA) and apigenin (>97%, Fluka) were used as HPLC references to identify this flavonoid and this iridoid isolated from MeOH extract. HPLC analysis was carried out using a LiChrospher^®^ 100 RP-18 column (4 mm × 250 mm, 5 µm) (Merck, Kenilworth, NJ, USA). The mobile phase consisted of two solvent reservoirs A (water/acetonitrile 97:3 mixture) and B (acetonitrile). The gradient system was as follows: 0–8 min, 100%–0% B; 9–12 min, 90%–10% B; 13–15 min, 80%–20% B; 16–20 min, 70%–30%, 21–25 min, 0%–100% B and 26–28 min 100%–0% B. The flow rate was 1 mL/min and the injection volume was 10 µL. The absorption was measured at λ = 205 nm for aucubin and λ = 340 nm for apigenin.

### 3.6. Bioactivity Assays

#### 3.6.1. Animals

Experiments were performed on male albino ICR mice (body weight range 30–35 g, Harlan, Mexico City, Mexico). The experimental protocol was approved by the local Ethics Committee on 23 December 2011, and received registration number R-2011-1701-86. All the experiments were carried out according to a protocol approved by the Institutional Research Committee in compliance with the Official Mexican Regulation (NOM-062-ZOO-1999). Minimum number of animals (*n* = 6) and duration of observation required to obtain consistent data were employed. Each animal was housed eight per cage, maintained under laboratory conditions at 25 °C, under 12-h light/12-h dark cycles, with lights turned on at 07:00 a.m. and having free access to water and standard food pellets (Harlan). The mice were allowed at least three weeks to adapt to the laboratory environment prior to initiating the experiments.

#### 3.6.2. Drugs

Imipramine hydrochloride (IMI, >99%, Sigma-Aldrich, St. Louis, MO, USA), diazepam (DZP, 98%, Sigma-Aldrich) and sodium pentobarbital (Pbi, Pfizer, New York, NY, USA) were used as antidepressant, sedative-anxiolytic and hypnotic drugs control, respectively. All of these treatments were administered *i.p.* 1% using Tween 20 solution (TW 2.5%, Merck) as vehicle.

#### 3.6.3. Experimental Design

Mice exposed to: pentobarbital-induced hypnosis (Pbi), open field (OFT) and elevated plus maze tests (EPM), were administered 1hr before each test when they were given the treatment by oral pathway (Ct 50, 100, 500, 750 mg/kg, and TW). The DZP (1.0 mg/kg) group received the drug its treatment *i.p.* 30 min before all tests. In case of, the forced swimming test (antidepressant activity, FST) the administration was 24, 18 and 1 h before the FST for Ct and 30 min by IMI (15.0 mg/kg, *i.p.*).

#### 3.6.4. Sedative Effect

##### Pentobarbital-Induced Hypnosis (Pbi)

A sub-hypnotic dose of sodium pentobarbital (30 mg/kg) was injected *i.p.* 30 min after administration of each treatment. The hypnotic effect was recorded showing the disappearance (latency) and the reappearance (duration) of the righting reflex. Hypnotic time was considered as the time interval between disappearance and reappearance of the righting reflex [12,13,14].

#### 3.6.5. Elevated Plus Maze (EPM)

The EPM test is the most widely used model for the anxiolytic-activity assessment of novel substances including herbal remedies in rodents [37,38]. The EPM apparatus was made of plexiglas and consisted of two open arms (30 cm × 5 cm) and two closed arms (30 cm × 5 cm) with 25 cm-thick walls. The arms extended from a central platform (5 cm × 5 cm), and the maze was elevated 38.5 cm from the room’s floor.

Each animal was placed at the center of the maze facing one of the enclosed arms. The number of entries and time spent on closed and open arms were recorded for 5 min. Entry into an arm was defined as the animal placed all four paws on the arm. All tests were recorded with a video camera. After each test, the maze was carefully cleaned with tissue paper soaked in a 10% ethanol solution. The percentage of number of entries to open arms (EOA) and the percentage of time spent in open arms (TOA) was registered.

#### 3.6.6. Open Field Test (OFT)

The open-field area was made up of acrylic transparent walls and a black floor (30 × 30 × 15 cm) divided into nine squares equal in area. The OFT was used to evaluate the exploratory activity of the animal [20]. All treatments were administered *i.p.*, 30 min before the test. The number of squares crossed (with the four paws) and the number of rearings were measured. The mice received the treatments 30 min prior to the experiment.

#### 3.6.7. Forced Swimming Test (FST)

The apparatus consisted of a glass cylinder (20 cm in height × 12 cm in diameter) filled with water (24 ± 1 °C) 15 cm deep. In the pre-test session, each animal was placed into the cylinder for 15 min, after this time, the mice were subjected to their respective treatment. After 24 h the mice were placed once again in the glass cylinder for 5 min. During the test session, a trained observer recorded the immobility time, when the mouse made no further attempts to escape, as well as the movements to maintain its head above water. It was suggested that immobility reflected a state of lowered mood in which the animals had given up hope of finding an exit and had resigned themselves to the experimental situation [22].

### 3.7. Statistical Analysis

All results data were performed with the SPSS 11.0 software program and based on analysis of variance (ANOVA) followed by the Dunnett test. A significant difference was established by comparison with control group, when the *p* value was <0.05.

## 4. Conclusions

In conclusion, the pharmacological and chemical study of *Castilleja tenuiflora* showed that the methanol extract displays sedative and hypnotic effects and contains principally one furofuran lignan named tenuifloroside (**1**). This is the first time that this chemical skeleton has been reported in the *Castilleja* genus. Caryoptoside, also isolated in this work, has not been identified in the *C. tenuiflora* species. Tenuifloroside and verbascoside were the major chemical constituents in the bioactive extract while the flavonoids apigenin and luteolin-5-methyl ether were the minor compounds. Considering that neuroprotective effect displayed by the complete extract may be due to only one of those identified metabolites or to a mixture of several components from this methanol extract, bioguided chemical analysis will continue.
